# Clinical Applications of Liquid Biopsy in Hepatocellular Carcinoma

**DOI:** 10.3389/fonc.2022.781820

**Published:** 2022-02-08

**Authors:** Jin-Cui Yang, Jun-Jie Hu, Yi-Xin Li, Wei Luo, Jin-Zhou Liu, Da-Wei Ye

**Affiliations:** ^1^ Cancer Center, Tongji Hospital, Tongji Medical College, Huazhong University of Science and Technology, Wuhan, China; ^2^ Department of Pain Management, The Second Affiliated Hospital of Guangxi Medical University, Nanning, China; ^3^ Department of Pancreatic-Biliary Surgery, Shanxi Bethune Hospital, Shanxi Academy of Medical Sciences, Tongji Shanxi Hospital, Third Hospital of Shanxi Medical University, Taiyuan, China

**Keywords:** clinical application, liquid biopsy, circulating tumor cells, circulating tumor DNA, exosome, hepatocellular carcinoma

## Abstract

Hepatocellular carcinoma (HCC) is a common malignant tumor with high mortality and poor prognosis in the world. The low rate of early diagnosis, as well as the high risk of postoperative metastasis and recurrence, led to the poor clinical prognosis of HCC patients. Currently, it mainly depends on serum markers, imaging examination, and tissue biopsy to diagnose and determine the recurrence and metastasis of HCC after treatments. Nevertheless, the accuracy and sensitivity of serum markers and imaging for early HCC diagnosis are suboptimal. Tissue biopsy, containing limited tissue samples, is insufficient to reveal comprehensive tumor biology information and is inappropriate to monitor dynamic tumor progression due to its invasiveness. Thus, low invasive diagnostic methods and novel biomarkers with high sensitivity and reliability must be found to improve HCC detection and prediction. As a non-invasive, dynamic, and repeatable detection method, “liquid biopsy”, has attracted much attention to early diagnosis and monitoring of treatment response, which promotes the progress of precision medicine. This review summarizes the clinical applications of liquid biopsy in HCC, including circulating tumor cells (CTCs), circulating tumor DNA (ctDNA), and exosome in early diagnosis, prognostic evaluation, disease monitoring, and guiding personalized treatment.

## Introduction

Hepatocellular carcinoma (HCC) is the main pathological type of primary malignant tumor of the liver, ranking as the sixth common cancer and the fourth leading cause of cancer-related death in the world (1). It mainly develops in the background of cirrhosis, resulting from hepatitis B and C virus infection, excessive drinking, or non-alcoholic fatty liver disease (2). There will be more than 1 million people to die in HCC by 2030 from the prediction of World Health Organization (WHO) (3). Surgical intervention including surgical resection and liver transplantation is the primary therapy to obtain satisfactory long-term results for HCC patients. However, due to the insidious onset and rapid progression of HCC, most patients have already reached the advanced stage of HCC during the first diagnosis and lost the opportunity to access surgical treatment (4). Currently, it mainly depends on serum markers, like alpha-fetoprotein (AFP), imaging examination as well as tissue biopsy to diagnose and determine recurrence and metastasis of HCC after treatments. Although clinical practice guidelines recommend that high-risk individuals undergo ultrasound (US) and serum AFP monitoring every 6 months (5), the sensitivity of this method for the detection of early-stage HCC is only 63% (6). Besides, elevated AFP may also be detected in some other diseases, for example, cirrhosis, hepatitis, intrahepatic cholangiocarcinoma, and metastatic colon cancer (7). AFP is no longer recommended as a part of the diagnostic evaluation by the latest American Association for the Study of Liver Disease (AASLD) guidelines (5). Therefore, low invasive diagnostic methods and novel biomarkers with high sensitivity and reliability must be found to detect the early-stage HCC and monitor the tumor recurrence.

**Figure 1:**
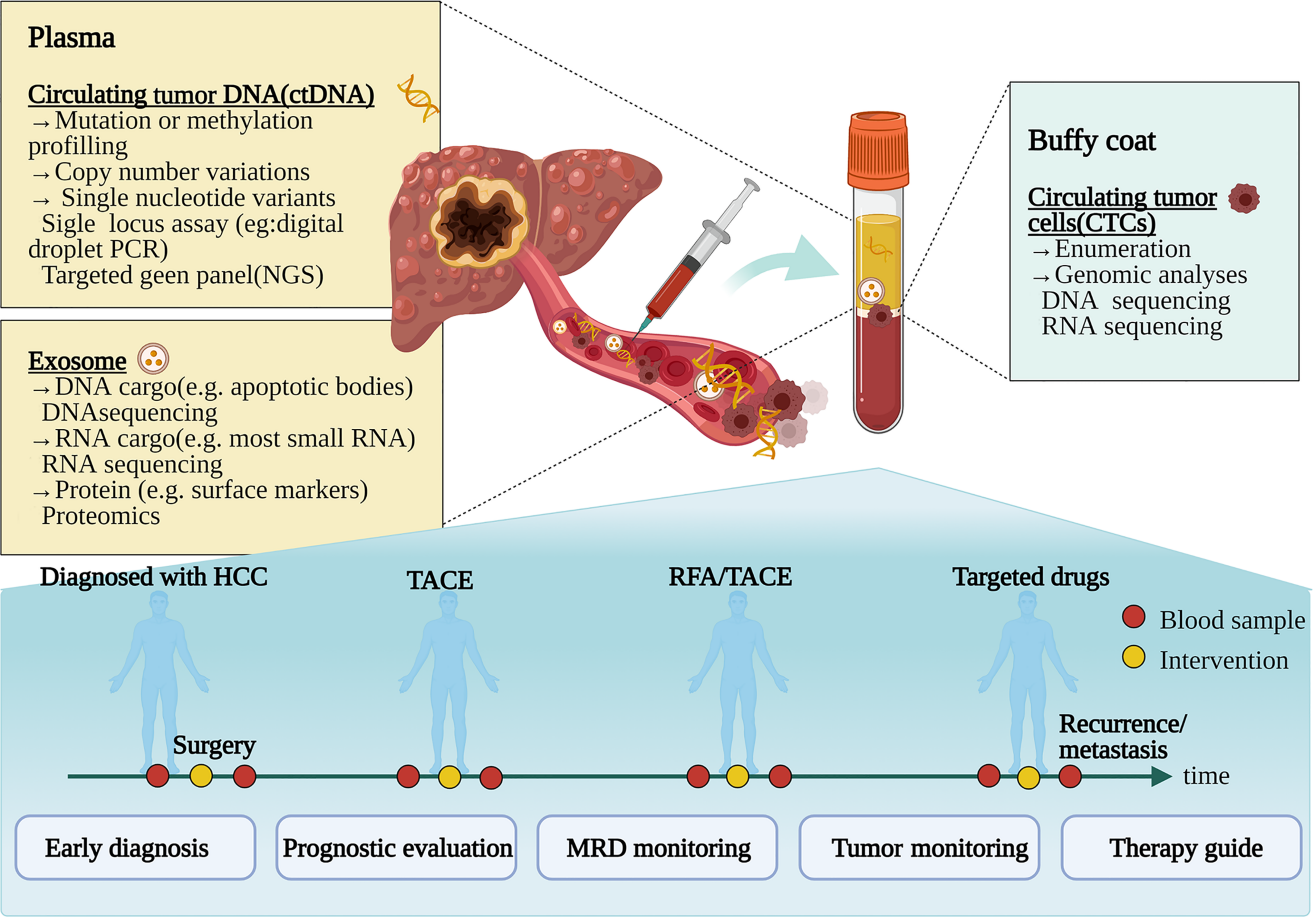
Clinical application pattern of liquid biopsy in patients with hepatocellular carcinoma. Tumor composition analyses, such as circulating tumor cells, circulating tumor DNA, and exosomes, are released by tumors to the bloodstream. During the various treatments such as surgery, transcatheter arterial chemoembolization (TACE), radiofrequency ablation (RFA), and targeting molecular treatment, liquid biopsy can be used for diagnosis, prognosis, and progress monitoring of HCC patients. NGS, next-generation sequencing; MRD, minimal/molecular residual disease. **Figure 1** created in BioRender (https://biorender.com/).

Liquid biopsy is a noninvasive, dynamic, and repeatable approach, which has emerged and shown significant prospects for HCC. Liquid biopsy obtains tumor-related information by collecting samples of body fluids such as blood and detecting circulating tumor cells (CTCs), circulating tumor DNA (ctDNA), and exosomes (8, 9) (**Figure 1**). Liquid biopsy has shown promising clinical application in several tumors, for instance, colorectal cancer (10), prostate cancer (11), lung cancer, and breast cancer (12). The increased studies of liquid biopsy in HCC have also emerged in recent years. Owing to HCC heterogeneity, only depend on a single biopsy might not be sufficient to reveal comprehensive tumor biology. Meanwhile, tissue biopsy is also inappropriate to monitor dynamic tumor progression as a routine practice due to its invasiveness. Fortunately, liquid biopsy can overcome these disadvantages to provide a real-time sample for the disease in a non-invasive and convenient way. As novel biomarkers, CTCs, ctDNA, and exosomes have made excellent progress in liquid biopsy of HCC (13–15). Analysis of specific gene mutations in ctDNA will help to better select treatment options and deal with drug resistance (14). This review summarizes the clinical applications of liquid biopsy in HCC, including CTCs, ctDNA, and exosomes in early diagnosis, prognostic evaluation, disease monitoring, and guiding personalized treatment.

## Circulating Tumor Cells (CTCS)

Thomas R. Ashworth first found CTCs from the peripheral blood in the 1860s (16). CTC is released into the blood circulation from primitive or metastatic tumor cells (17). Tumor cells migrate into the bloodstream becoming CTCs by secreting matrix metalloproteinase to break the basement membrane (18). Then they can invade different parts of the body through blood circulation, thus being vividly described as “seeds” of the tumors (14). A crowd of tumor cells is released into circulation every day. However, less than 0.01% of CTCs could eventually survive and lead to a fatal metastasis (19). The half-life of CTCs is 1-2.4h, most CTCs introduced into the circulation are eliminated by shear stress, immune attack, and anoikis (20, 21). At present, the CTC defined by the CellSearch™ system is an accepted standard: CTC is a kind of epithelial cell with an intact nucleus, which is positive for EpCAM and/or cytokeratin 8, 18, and 19, but do not express CD45 (13). Meanwhile, CTCs gain the mesenchymal features by downregulating epithelial cell adhesion molecule EpCAM expression (22), thus enhancing the ability to enter the lymph vascular system (23), this process is called the epithelial-to-mesenchymal transition (EMT). In the process of tumor metastasis, EMT is triggered as a consequence of the interaction between various cellular signaling pathways, like Notch, Wnt, PDGF, TGF-β, Akt, and NF-κB (24). There are three subtypes of CTC in the process of EMT, including epithelial CTCs, mesenchymal CTCs, and mixed CTCs, which have both epithelial and mesenchymal CTCs (25). Also, CTCs may aggregate to form CTC clusters with or without leukocytes, platelets fibroblasts, or endothelial cells and move together in the bloodstream. CTC clusters are rare compared to individual CTC, but they tend to have increased survival ability and metastatic potential (26). Because of its short half-life and limited detection methods, the importance of CTC clusters may be underestimated.

Several detection and isolation techniques of CTCs have been produced, which are mainly divided into biological and physical methods. CTCs can be separated from other nucleated cells or normal epithelial cells by utilizing their distinct physical properties, and molecular characteristics, such as density, measurement, electric charge, deformation ability, and transfer capacity, as well as biological parameters, including cell surface markers, or combining both characteristics (13). The widely used technologies for CTCs detection are fluorescence *in situ* hybridization (FISH), reverse transcriptase-polymerase chain reaction (RT-PCR), immunofluorescence (IF), next-generation sequencing (NGS), and microfluidic-based techniques (14). CellSearch™ system that targets EpCAM to quantify CTCs remains the first and only clinically verified technology for enrichening and counting CTCs approved by the Food and Drug Administration (FDA). However, there is only a small portion of the HCC patients with EpCAM positive, and this method may underestimate the number of CTCs due to the existence of EMT. Hence, it is imperative to explore novel technologies with high accuracy to improve the capture rate of CTCs and expand the clinical application.

## Clinical Applications of CTCS IN HCC

CTC has a strong prognostic effect, especially after resection, it also shows potential in monitoring the progress of HCC and guiding treatment (14). Decades of CTCs research have made great progress in the clinical application of HCC (**Table 1**).

**Table 1:** Clinical applications of CTC in HCC.

Region	Patient	Method	CTC markers	Function of the marker	Positive rate	Ref.
Prognostic evaluation						
China	123	CellSearch™	EpCAM	Epithelial marker	67%	(27)
China	49	negative enrichment qRT-PCR	EpCAM, CD4+CD25+Foxp3Treg cells	EpCAM: epithelial marker; Treg cells: immune escape	35%	(28)
United Kingdom	69	ImageStream	AFP, EpCAM, GPC3, DNA- PK	AFP, GPC3: related biomarkers of HCC; DNA- PK: candidate biomarker for treatment stratification in HCC; EpCAM: epithelial biomarker	65%	(29)
China	47	flow cytometry qRT-PCR	MAGE-3, Survivin, CEA	MAGE-3, Survivin: metastasis-associated markers; CEA:carcinoembryonic antigen	Not applicable	(30)
China	139	CellSearch™	EpCAM	Epithelial marker	44%(Pre) 54%(post)	(31)
China	112	CanPatrol™	EpCAM, CK, Vimentin, Twist	EpCAM, CK: epithelial marker; Vimentin, Twist: mesenchymal marker	90%	(32)
Germany	57	CellSearch™	EpCAM	Epithelial marker	16%	(33)
China	89	CellSearch™	EpCAM	Epithelial marker	56%	(34)
United States	20	CellSearch™	EpCAM	Epithelial marker	40%	(35)
China	42	CTC-Chip	EpCAM	Epithelial marker	60%	(36)
China	195	CanPatrol™	CK, EpCAM, Twist, Cadherin, Vimentin, AKT2	EpCAM, Cadherin,CK: epithelial marker; Twist, Vimentin, Snail, AKT2: mesenchymal marker;	95%	(37)
Germany	59	CellSearch™	EpCAM	Epithelial marker	31%	(38)
China	299	negative enrichment qRT-PCR	EpCAM^mRNA+^	Epithelial marker	43%	(39)
China	73	CellSearch^TM^ qRT-PCR	EpCAM, E-cadherin, N-cadherin, Vimentin, Snail, Slug	EpCAM, E-cadherin: epithelial marker; N-cadherin, Vimentin, Snail, Slug: mesenchymal marker	68%(PV) 45%(PA) 81%(HV) 40%(IHIVC) 59%(PoV)	(40)
China	14	SE-Ifish	Aneuploid chromosome 8	genomic instability	8% (EpCAM+ CTSC) 86% (EpCAM- CTC)	(41)
Korea	105	Tapered slit filter, immunofluorescence	CK, CD45	CK: epithelial marker; CD45: leukocyte marker	24%(ΔCTC>0)	(42)
China	137	CellSearch™	EpCAM	Epithelial marker	34%	(43)
China	214	CanPatrol™ RNA-ISH	CD45, EpCAM, DAPI CK8/18/19, vimentin/twist,	EpCAM, CK: epithelial marker; Twist, Vimentin, mesenchymal marker; CD45: leukocyte marker; DAPI: nuclei marker	42%	(44)
Monitoring and guide therapy						
China	136	CanPatrol™	CD45, EpCAM, CK8/18/19, vimentin, twist	EpCAM, CK: epithelial marker; Twist, Vimentin, mesenchymal marker; CD45: leukocyte marker	92%	(45)
China	30	PowerMag negative selection system	EpCAM, Hoechst, CD45,	EpCAM: epithelial marker; CD45: leukocyte marker; Hoechst: nuclei marker	100%	(46)
China	109	immunofluorescence staining	pERK	Sorafenib-related targets	93%	(47)
United States	6	IFC scRNA-seq	ASGPR1, pan-CK, GPC3, EPCAM, CD45	EpCAM, pan-CK: epithelial marker; CD45: leukocyte marker; GPC3: related biomarkers of HCC; ASGPR1: expressed in hepatocytes	67%	(48)

qRT-PCR, quantitative real-time polymerase chain reaction; PV, peripheral vein; PA, peripheral artery; HV, hepatic veins; IHIVC, infrahepatic inferior vena cava; PoV, portal vein; IFC, Imaging Flow Cytometry; scRNA-seq, Single-cell RNA sequencing.

### Prognostic Evaluation

Current data do not provide clear evidence to support CTCs as an early-stage HCC diagnostic tool (14, 49). But as for prognosis, a great deal of research supports its prospect in predicting therapeutic outcome and monitoring disease progression, particularly after resection (3). Sun et al. (27) applied CellSearch™, for the first time, to capture and analyze EpCAM+ CTCs in 123 patients before the resection of HCC and 1 month thereafter. They found that EpCAM+ CTCs≥2 per 7.5 ml of blood were proved to be the strongest prognosticator, especially in the subgroup of patients whose AFP level was lower than that of 400ng/ml. Additional similar studies proved that the expression of EpCAM+ CTCs in HCC patients showed a significant positive correlation to the serum AFP level (35), BCLC stage (38) and was associated with vascular invasion (40), disease progression (35), higher recurrence rate (33), and shorter disease-free survival (DFS) and overall survival (OS) (31). Prospective research on 135 HCC patients demonstrated that surgical resection of tumors reduced the quantity of CTC. They suggest that the postoperative CTC continued to be at a higher level (≥5), which has a better predictive validity for prognosis than AFP>400cmg/L and tumor diameter>5cm (43).

However, EpCAM was positive in less than 20% of HCC cases (50), and some of them also develop into EMT. This will reduce the detection rate of EpCAM+CTCs and limit its clinical application. The Canpatrol™ CTC analysis platform was developed for EMT, which uses microfiltration and various markers to characterize CTCs. This technology not only uses epithelial markers (EpCAM, CK8/9/19) but also adds mesenchymal markers (Vimentin and Twist) (51). Qi et al. (32) used the CanPatrol™ CTC-enrichment technique in 112 HCC patients with a positive rate of more than 90%, even for early-stage diseases. Before resection, the number of CTCs≥16 and the percentage of mesenchymal CTCs≥2% were greatly correlated with intrahepatic recurrence and distant lung metastasis. Some negative enrichment methods and combinations of different markers or technologies of CTCs have also been established to optimize the platform (29, 46, 52).

Except for the number of preoperative CTCs, the forms of CTCs also influenced the prognosis of HCC patients. Mesenchymal CTCs have more invasion and metastatic potential. Bai et al. (53) showed that high expression of CXCR4 protein in mixed CTCs was more common, which might be associated with the progression and metastasis. Another study found that the proportion of mixed and mesenchymal CTCs was also a prognostic indicator of HCC (37). In a prospective study, Sun et al (40). examined CTCs at five important sites of blood vessels in HCC patients, and showed that the presence of multi-vascular CTC cluster could prognosticate recurrence and metastasis. The CTC clusters are also been reported in some studies. Gkountela et al. (54) showed that the specific hypermethylation of the binding sites for transcription factors related to stemness and proliferation in the CTC cluster promoted metastasis. The CTC-associated white blood cell clusters of peripheral blood in patients with HCC were also related to DFS and OS (44).

### Tumor Monitoring And Guiding Personalized Therapy

CTCs may serve as an index for long-term monitoring of HCC, and its dynamic changes reflected the therapeutic response treated with locoregional therapies, such as transcatheter arterial chemoembolization (TACE), radiotherapy, and radiofrequency ablation (55–57). Rau et al. (46) used a negative selection method for the enrichment of CTCs and found that the analyzes of CTCs at a different time during the treatment is helpful to dynamically monitor the progression of HCC patients, especially those without elevated serum AFP levels. Fan et al. (58) investigated a new technique which was combined flow cytometry *in vivo* with orthotopic tumor models and showed that the number of CTCs decreased significantly after resection and early metastases were also reduced. The tumor size and the number of distant metastases of HCC were in accord with the dynamic change of CTCs. Moreover, Zhang et al. (59) used microfluidic chip technology to obtain CTCs and discovered that the number of spheroids formed by CTCs substantially reduced after treated with sorafenib or oxaliplatin. This reflects the potential of CTCs in the analysis of sensitivity and drug resistance of chemotherapeutic drugs.

Identifying CTCs that express specific tumor markers and drug targets enable doctors to better guide personalized therapy. In 2016, Li et al. (47) indicated that CTCs can replace tumor tissue to characterize the expression of pERK/pAkt. The pERK+/pAkt- CTCs were most sensitive to sorafenib, which is helpful to choose the appropriate treatment scheme. In recent years, immunotherapy is a research hotspot, and immune checkpoint inhibitors, including Programmed cell death protein-1 (PD-1) and programmed death-ligand 1 (PD-L1), are promising in the treatment of advanced HCC (60). Winograd et al. (61) firstly evaluated PD-L1+ CTCs in HCC. They reported that PD-L1+ CTCs could serve as a predictor of immunotherapy. Because 3 of all 6 patients who received anti-PD1 therapy showed responses to treatment and had PD-L1+ CTCs (61). In 2020, the same team conducted a larger, prospective cohort to enumerate/phenotype CTCs. They showed that PD-L1+ CTCs mainly present in advanced HCC, and related to beneficial therapeutic responses of patients with HCC who received anti-PD-1 therapy (62). The expression of PD-L1 varies with immune statuses, treatment response, and disease progression. Precise assessment of PD-L1 expression in CTCs could be used to evaluate and monitor the immune status of tumor cells in real-time.

## Circulating Tumor DNA (ctDNA)

When it comes to ctDNA, it is inevitable to mention circulating cell-free DNA(cfDNA), that derives from lymphocytes or dying benign host cells (63). It is generally believed that ctDNA is the fragmented DNA shedding from necrotic and apoptotic tumor cells, occupying only a small portion of total cfDNA (14, 64). The mechanism of how ctDNA is released into the bloodstream is still unclear. It might be associated with apoptosis and necrosis (65), and exposure to intermittent hypoxia is likely to promote ctDNA fall off into the circulation (66). At present, there are still some difficulties in separating ctDNA from cfDNA using the existing technology. Methylation of ctDNA and cfDNA is research hotspots (14). DNA methylation is involved in the epigenetic regulation of gene expression and often leads to gene silencing. The ctDNA has the molecular characteristics of methylation changes, and it has been found that the tumor DNA methylation profiles of HCC are highly correlated with the paired plasma ctDNA (67). Methylation changes in ctDNA usually occur early in carcinogenesis (14), therefore, the detection of methylation genes in ctDNA has a certain clinical potential in HCC (67–69). Moreover, ctDNA comprises a complete tumor genome, including variants derived from a plurality of independent tumors, so ctDNA has a greater advantage in overcoming tumor heterogeneity than single tissue biopsy.

Since only 10 ng cfDNA can be extracted per milliliter of blood (70), the detection method of ctDNA should be highly sensitive and specific. Various methods can be selected according to different detection purposes. The ctDNA carries tumor-specific information in terms of genetic or epigenetic alterations, like methylation changes, single-nucleotide variants (SNVs), and copy number variations (CNVs). There are two types of analytical techniques based on ctDNA: quantitative detection (measure the number of ctDNA) and qualitative detection (detect tumor-specific genetic aberration). The length of ctDNA fragments is less than 167 base pairs, approximately the size of 1 nucleosome (71). Moreover, the half-life of ctDNA is short, usually, no more than 2 hours, which can relatively accurately reflect the real-time change of the tumor burden during cancer therapy. The ctDNA carries the same genetic mutation as the primitive tumor cell, thus the qualitative and quantitative analysis of ctDNA is mainly based on detecting the aberrations in cfDNA. Digital PCR (dPCR) and NGS are two popular methods nowadays techniques to detect ctDNA.

## Clinical Application OF ctDNA IN HCC

The ctDNA contains genomes derived from multiple independent tumors (72), thus, detecting ctDNA is expected to overcome temporal and spatial heterogeneity of tumor tissues. We summarized the clinical application of ctDNA in patients with HCC (**Table 2**).

**Table 2:** Clinical applications of ctDNA in HCC.

Region	Patient	Target site	Methods	Function of the gene	Ref.
Early detection and prognosis					
China	37 HCC 33 healthy	DBX2, THY1	Targeted bisulfite sequencing	Hypermethylation of DBX2, THY1 may result in HCC development	(73)
Hong Kong	26 HCC 32 healthy	Hypomethylation, CNAs	Massively parallel bisulfite sequencing	/	(74)
United States	66 HCC 43 benign chronic liver diseases	INK4A	Pyrosequencing and MSP	Promoter hypermethylation of INK4A leads to loss of p16 expression	(75)
China	121 HCC 37 chronic hepatitis B 31 healthy	MT1M, MT1G promoter	MSP	Methylation of MT1M and MT1G promoters is associated with vascular invasion or metastasis	(76)
China	100 HCC 29 healthy	HOXA9	MSP, bisulfite sequencing, and Q-MSP	Hypermethylation of HOXA9 may be present in precancerous lesion during carcinogenesis	(77)
China	1098 HCC 835 healthy	BMPR1A, PSD, ARHGAP25, KLF3, PLAC8, ATXN1, Chr 6:170, Chr 6:3, ATAD2, Chr 8:20	Targeted bisulfite sequencing	/	(67)
Taiwan	180 HCC	APC, COX2, RASSF1A miRNA	qMSP	Hypermethylation of RASSF1A suggests the early stage of HCC. Hypermethylation of APC and COX2 is associated with liver carcinogenesis	(78)
Taiwan	237 HCC 257 controls	TBX2	Pyrosequencing assay, Real-time PCR	Hypermethylation of TBX2 is associated with increased HCC risk	(79)
France and Germany	98 HCC 191 cirrhosis	SEPT9	MSP	Hypermethylation of SEPT9 is associated with liver carcinogenesis	(68)
United States	116 HCC 81cirrhosis 98 healthy	HOXA1, EMX1, AK055957, ECE1, PFKP, CLEC11A	qMSP	/	(69)
China	1204 HCC 392 CH/cirrhosis 958 healthy	5hmC modifications	5hmC-Seal technique	Serve as ideal markers for specific gene/locus activation in chromatin	(80)
China	508 HCC 2250 cirrhosis 476 healthy	5-hmc, NF, 5′end motif, fragmentation	NGS	/	(81)
	ctDNA mutation				
United States	66 HCC 35 cirrhosis 41HCV-related chronic hepatitis	hTERT	real-time PCR	The amount of hTERT gene in plasma served as serves as a surrogate of cfDNA	(82)
China	48 HCC	TP53, CTNNB1, TERT	Droplet digital PCR	Mutation of TP53 and CTNNB1 suggests the occurrence of HCC; TERT promoter mutation is an early event in liver carcinogenesis;	(83)
China	41 HCC	TERT, CTNNB1, TP53	MiSeq sequencing	Mutation of TP53 and CTNNB1 suggests the occurrence of HCC; TERT promoter mutation is an early event in liver carcinogenesis;	(84)
China	65 HCC 70 non- HCC 331 at risk patients	TP53, CTNNB1, AXIN1, the TERT promoter, HBV insertion site, AFP, DCP	HCCscreen	AXIN1 mutation is associated with HCC	(85)
China	384 HCC	SCNA	WGS	/	(86)
	Monitoring and guide therapy				
China	34 HCC	SNVs, CNVs	Target sequencing Whole exome sequencing	/	(87)
United States	14 HCC	TP53, CTNNB1, PTEN, CDKN2A, ARID1A, MET; CDK6, EGFR, MYC, BRAF, RAF1, FGFR1, CCNE1, PIK3CA, ERBB2/HER2	NGS	/	(88)
United States	26 HCC	TP53, CTNNB1, ARID1A	NGS	Mutations of TP53, CTNNB1 and ARID1A are associated with treatment response	(89)

Q-MSP, Quantitative methylation-specific PCR; MSP, methylation-specific PCR; qMSP, real-time quantitative methylation-specific PCR; WGS, whole-genome sequencing; NGS, next-generation sequencing; SNVs, single nucleotide variants; CNVs, copy-number variants; SCNA, somatic copy number aberration.

### Early Diagnosis And Prognostic Evaluation

Epigenetic changes induced by DNA methylation and DNA methylation are involved in the process of tumor occurrence and development (90). The methylation pattern of ctDNA has tremendous potential in the early diagnosis of HCC patients. Changes in methylation of multiple genes in plasma/serum, such as p15, p16, GSTP1, INK4A, RASSF1A, and so on, have been confirmed in many studies to distinguish HCC from controls. The methylation characteristics of tumor DNA in HCC are highly correlated with paired plasma ctDNA (67), so some biomarkers of DNA methylation in HCC may also be used in ctDNA. Kotoh et al. (91) developed a methylated SEPT9 assay in HCC, with 63.2% sensitivity and 90.0% specificity. They pointed out that combined diagnosis with AFP can improve the diagnosis rate of early-stage HCC. Yan et al. (92) proposed the HCC index, which was a combined diagnostic model of cfDNA, age, and AFP. The HCC index was more accurate in HCC diagnosis than cfDNA or AFP, alone. Currently, Chen et al. (81) conducted large-scale, multi-center research and constructed a diagnostic model based on HIFI (5-Hydroxymethylcytosine/motIf/Fragmentation/nucleosome footprInt) method, playing a key role in differentiating HCC from non-HCC. Both the test set and the verification set had a sensitivity and specificity of more than 95%.

In addition to the role of diagnosis, ctDNA methylation can also be served as a prognostic indicator. The study of Kotoh et al. also showed that the copy number of methylated SEPT9 was related to BCLC stage, macrovascular invasion, tumor number, and size (91). Li et al. (93) indicated that IGFBP7 promoter methylation was significantly related to OS and early tumor recurrence after hepatectomy. In a large-scale study involving 1098 HCC patients, a diagnostic prediction model was constructed by screening 10 overlapping markers of cfDNA methylation using Random Forest and Least Absolute Shrinkage and Selection Operator (LASSO) methods. The diagnostic sensitivity and specificity of this model were 85.7% and 94.3%, respectively (67). Kisiel et al. (69) identified 6 best-methylated DNA markers (MDMs) in HCC, that include ECE1, HOXA1, CLEC11A, AK055957, PFKP and EMX1, and combined Phase I Pilot, and Phase II Clinical Validation. In the diagnosis of HCC, the area-under-the-receiver-operating-curve (AUC) of this 6-marker MDM panel was 0.96, the sensitivity was 95%, and the specificity was 92%. Moreover, elevated levels of cfDNA are a positive correlation with poor prognosis. The existence and quantity of ctDNA are usually determined by detecting the mutation of cfDNA. Some hot mutants, such as TP53, CTNNB1, and TERT, are usually selected to detect ctDNA aberrations (14). Ren N et al. (94) found that allelic imbalance at D8S258 in circulating plasma DNA was also related to the poor prognosis of HCC. A recent study focused on SNVs of ctDNA and showed that the existence of MLH1 SNV, coupled with elevated ctDNA levels, can predict poor OS of HCC patients (95). In short, the different mutants and epigenetic modifications in ctDNA have significance in early diagnosis and prognostic outcomes of HCC.

### Tumor Monitoring and Guiding Personalized Therapy

Considering that liver biopsy is invasive and unnecessary in advanced HCC, ctDNA can be a reliable biomarker for dynamically monitoring tumor progression and assessing the treatment efficacy. These include identifying new mutations that drive acquired drug resistance and capturing heterogeneity between tumors. According to the study of Park et al. (96), the high level of cfDNA after radiotherapy was related to the poor outcome of treatment. Thus, ctDNA can be used as an indicator to evaluate the curative effect after radiotherapy. Recently, Zhao et al. (97) prospectively enrolled 42 patients with unresectable liver cancer. They found that TP53 mutation was related to disease progression and interventional treatment was more effective in patients without TP53 mutation. The follow-up study showed that plasma levels of CNVs and SNVs in ctDNA dynamically correlated with patients’ tumor burden in HCC (80). They decreased after surgery and increased in cases with tumor recurrence, showing that ctDNA was a feasible biomarker to monitor treatment response (80). Cai et al. (87) collected the information of postoperative ctDNA and protein biomarkers which included AFP, AFP-L3, and des-gamma-carboxy prothrombin (DCP), then evaluated the results with corresponding MRI scan images during follow-up. They confirmed that both SNVs and CNVs of ctDNA could dynamically monitor the tumor load of HCC. The combination of ctDNA and DCP could improve the detection rate of minimal/molecular residual disease (MRD) in patients undergoing hepatectomy (87).

On other hand, the detection of ctDNA mutations can guide the choice of treatment. Ikeda et al. (98) used digital ctDNA sequencing to evaluate the mutant associated with wild-type allele fraction in 14 advanced HCC patients. The level of DCP and AFP decreased with the treatment of palbociclib (CDK4/6 inhibitor) and celecoxib (COX-2/Wnt inhibitor) after two months in a patient with CDKN2A-inactivating and CTNNB1-activating mutation. And AFP declined by 63% in another patient treated with sirolimus (mechanistic target of rapamycin inhibitor) and cabozantinib (MET inhibitor). This patient had PTEN-inactivating and MET-activating mutations of ctDNA (98). Compared with tissue biopsies, cfDNA identified clinically related drug resistance changes more frequently in a prospective cohort study of patients with gastrointestinal tumors. In 78% of cases, cfDNA could detect drug resistance gene mutations that were not found in matched tumor biopsies (99). A recent study reported that the sequential mutation profiling of ctDNA can be used for molecular detection of drug resistance in HCC (100). The gene mutation of PI3K/MTOR pathway was associated with the worse PFS in HCC patients receiving tyrosine kinase inhibitors but was not associated with immunosuppression therapy (100). It shows that monitoring the mutation of the drug target gene or drug resistance gene in advanced patients can better guide the choice of personalized treatment. All in all, genomic profiles of patients with HCC can be obtained from ctDNA, which can guide the treatment to some extent.

### Extracellular Vesicles (EVs): Exosomes

Exosomes were thought to originate from mature sheep reticulocytes (101). Exosome belongs to extracellular vesicles (EVs), which is a nano-sized phospholipid bilayer membrane vesicle and responsible for intercell communication (102). It is secreted by living cells and formed by the separation of intracellular poly vesicles with cell membranes in the process of being released out of the cell (103). The component transported in exosomes contains several molecular biomarkers including proteins, RNA, DNA, which range in size from 50 to 140 nm (88, 104). Currently studies suggest that the cargos carried by exosomes associated with HCC, including non-coding RNAs(ncRNAs), messenger RNAs (mRNAs), and proteins, can serve as potential biomarkers for the clinical application of HCC (105). They transferred to target cells by exosomes, affecting drug resistance, tumor angiogenesis and metastasis (106–108). Exosomes show significant superiority in liquid biopsy. Exosomal substances are hard to be degraded due to the protection of phospholipid bilayer membrane (15). Its high biological stability improves the clinical applicability of exosomes, which can not only cut down the cost of short-term sample preservation but also reduce the challenges of transportation (109). Moreover, exosomes carry biological information from parental cells, so they are more typically than cfDNA (110). Exosomes are bound up with the growth and metastasis of HCC (108), tumor angiogenesis (111), and immune regulation (112).

The capture and enrichment of exosomes require the identification of their markers, such as heat shock protein 70 (HSP 70), CD9, CD63, CD81, and ALIX (113). There are some common technologies used for exosomes isolation according to their composition or physical properties, such as ultracentrifugation (UC), transmission electron microscopy (TEM), magnetic associated cell sorting (MACS), filtration, polymer-based precipitation, nanoparticle tracking analysis, fluorescence, colorimetric ELISA assays, and size exclusion chromatography (SEC). However, the eventual clinical utility of exosomes is still in its preliminary stages and needs more validation.

## Clinical Application OF Exosome IN HCC

Exosomes are related to the establishment of the tumor microenvironment and participate in the occurrence, development of HCC (114). Substantial researches have demonstrated that exosomes play a part in clinical applications of HCC over the years (**Table 3**).

**Table 3:** Clinical applications of exosomes in HCC.

Region	Patients	Method	Target	Function of the Cargo	Reference
**Korea**	20 HCC 20 CH 20 cirrhosis	Ultracentrifugation	miR-18a, miR-101, miR-106b, miR-122, miR-195, miR-221, miR-222, miR-224	/	(115)
**Korea**	84 HCC 26 CH 32 cirrhosis 26 healthy	ExoQuick™ Exosome Precipitation Solution	The panel based on miR-4661-5p	Immune escape	(116, 117)
**Korea**	90 HCC 60 CLD 28 healthy	Ultracentrifugation	miR-10b-5p, miR-215-5p	Invasion and metastasis of HCC	(118)
**Spain**	29 HCC 32 healthy 43 CCA 30 PSC	Filtration, Ultracentrifugation,	FIBG	unknow	(119)
**China**	74 HCC 26 Cirrhosis 34 CHB 72 healthy	ExoQuick™ Exosome Precipitation Solution	lncRNAs X-inactive-specific transcript	Regulate proliferation and metastasis of HCC	(120)
**China**	115 HCC 156 CHB 85 LC 120 healthy	Total Exosome Isolation Kit	ENSG00000258332.1, LINC00635, AFP	Regulate proliferation and metastasis of HCC	(121)
**China**	29 HCC 37 healthy and benign hepatomas	ExoQuick™ Exosome Precipitation Solution	SMAD3	Promoted cell adhesion	(122)
**China**	50 HCC 40 cirrhosis	Ultracentrifugation, filtration, and precipitation	A panel combining miR-122, miR-148a, and AFP	Inhibit proliferation and multidrug resistance	(123)
**Korea**	79 HCC	ExoQuick™ Exosome Precipitation Solution	miRNA-21 lncRNA-ATB	Regulate proliferation, invasion and metastasis of HCC	(124)
**China**	82 HCC 47 healthy	ExoQuick-TC exosome precipitation solution	circPTGR1	Promote metastasis of HCC	(125)
**China**	240 HCC	ExoQuick™ Exosome Precipitation Solution	circUHRF1	Immune escape	(126)
**China**	71 HCC 40 HD	Ultracentrifugation	A panel combining circ_0004001, circ_0004123, and circ_0075792	Regulate the proliferation, migration, and invasion of HCC	(127)
**Korea**	32 HCC 28CH 35 cirrhosis	ExoQuick™ Exosome Precipitation Solution	LINC00853	unknow	(128)
**China**	122 HCC 43 cirrhosis	Ribo™ Exosome Isolation Reagent	Lnc85	Inhibit proliferation and migration of HCC	(129)
**India**	38 HCC 35 CH 25 cirrhosis	ExoEnrich™ instant exosome isolation kit and immunoaffinity capture (anti-ASGR2)	A panel combining miR-10b-5p, miR-221-3p, miR-223-3p, and miR-21-5p	/	(130)

CCA, Cholangiocarcinoma; PSC, primary sclerosing cholangitis; CHB, chronic hepatitis B; LC, liver cirrhosis.

### Early Diagnosis and Prognostic Evaluation

Exosomes contain bioactive compounds, such as RNA, DNA, proteins, and cholesterol (15), some of which can be expressed uniquely by tumor cells. MicroRNAs (miRNAs), lncRNA and circular RNAs (circRNA) have been described in many reports as noninvasive biomarker for better prediction and prognosis of HCC progression. The miRNAs, a class of conserved RNAs (usually 22-25 nucleotides), can inhibit the translation of mRNA or promote the degradation of mRNA by combining with their response element on the 3-untranslated region of the target mRNA (107). Exosomes can actively secrete miRNAs to regulate the progress of HCC (15, 131). Sohn et al (115) found that serum exosomal miR-222, miR-221, and miR-18a in patients with chronic hepatitis or cirrhosis were significantly lower than that in HCC patients. Wang et al. (132) showed that the serum exosomal miR-21 also elevated in HCC patients, which could be used to distinguish HCC from people and chronic hepatitis B patients. Ghosh et al. (130) found that the combination of four miRNAs (miR-221-3p, miR-223-3p, miR-10b-5p and miR-21-5p) showed good diagnostic ability in patients with low expression of AFP. Currently, Cho et al (116) showed that serum exo-miR-10b-5p had great potential for early diagnosis of HCC with a sensitivity of 90.7%, specificity of 75.0%, and the AUC was 0.934. The same team developed a panel that included exo-miR-4746-5p and exo-miR-4661-5p for the early diagnosis of HCC. The sensitivity, specificity, and AUC were 81.8%, 91.7%, and 0.947 respectively (118). Along with the miRNA, lncRNA and cirRNA in exosomes have also shown potential in the early detection of HCC. The expression of LINC00853, lnc85, ENSG00000248932.1, ENST00000440688.1 and ENST00000457302.2, have shown promise for the tumorigenesis prediction (128, 129, 133). Xu et al. (121) suggested that AFP combined with serum exosomal LINC00635 and ENSG00000258332.1 could discriminate HCC from chronic hepatitis B, gaining an AUC of 0.894. By comparing the level of exosomal Trna-derived small RNA (tsRNA) between the healthy and patients with liver cancer, Zhu et al. (134)found a significant increase of tsRNAs in plasma exosomes of liver cancer patients, which provided new insight into the HCC diagnostic potential of the exosome. Currently, a study suggested that the combination of three circRNAs, including circ_0004001, circ_0004123 and circ_0075792, has been served as a valuable diagnostic biomarker for HCC (127).

In addition to ncRNA, proteins are also used as biomarkers of HCC. Compared with miRNA, the study of protein in exosomes is a relatively less explored direction to detect HCC. According to a study that the proteomes of HCC, cirrhosis and healthy people have different compositions (135). Arbelaiz et al. (119) suggested that the differentially expressed exosomal proteins might be a kind of potential biomarkers for differential diagnosis. Mass spectrometry was used to detect the proteomes of exosomes in patients with HCC, primary sclerosing cholangitis, and cholangiocarcinoma. Some of these proteins including LG3BP and FIBG demonstrated superior diagnostic ability to AFP. The AUC of LG3BP for identifying HCC patients from the healthy was 0.904, and the AUC of FIBG for distinguishing intrahepatic cholangiocarcinoma from HCC was 0.894 (119). Interestingly, LG3BP and PIGR can promote the transformation, invasion, and proliferation of tumor cells, which are associated with poor prognosis (136). Fu et al. (122) found that patients with advanced HCC had higher levels of Smad3 in exosomes and suggested that combined AFP and detection of exosomes containing SMAD3 can improve the diagnosis of HCC.

Exosomes also have great potential in terms of the prognosis of HCC. Lee et al. (124) demonstrated that circulating exosomal miRNA-21 and lncRNA-ATB were associated with the T stage, the TNM stage, and portal vein thrombosis. The HCC patients who had the level of exosomal miRNA-21≥0.09 and lncRNA-ATB ≥0.0016 tend to have significantly lower OS and PFS (P<0.05). The high level of LINC00635 and ENSG00000258332.1 in HCC was related to lymph node metastasis, TNM stage, and OS (121). Some studies showed that the lower levels of exosomal miR-125b (137) and miR-638 (138) predicted poor prognosis of HCC patients. The abundance of exosomes containing SMAD3 was negatively correlated with DFS in postoperative patients of HCC (122). Moreover, circUHRF1 might be associated with resistance to anti-PD1 immunotherapy in HCC patients (126). Luo et al. (139) measured the level of exosomal circular RNA (circAKT3) from 124 patients with HCC and 100 healthy controls and found that patients with high exosomal circAKT3 tend to have a higher risk of recurrence and death.

## Conclusion and Perspective

With the continuous enrichment of molecular tumor information and the frequent breakthroughs of molecular technology, precision oncology has revolutionized the field of medicine. Noninvasive liquid biopsy has competitive advantages for accurate diagnosis and individualized management (140). The detection and analysis of CTCs, ctDNA, and exosomes provide a promising strategy for early diagnosis, prognostic evaluation, the guidance of treatment, and monitoring of MRD and recurrence (**Figure 1**). DNA methylation is a widely used epigenetic biomarker, and the analysis of ctDNA methylation has the potential of early diagnosis of high-risk patients with HCC. This may soon become an alternative method for long-term monitoring of HCC, and be a supplement to the existing clinical detection methods. The molecular characterization of ctDNA and CTCs are, at least in part, dependent on tumor burden, so they may be more useful in intermediate or advanced settings in prognosis or predicting treatment response. In general, the increase of CTCs level after treatment indicates tumor recurrence and reduced survival. Further studies of CTC and ctDNA will better understand the emergence of resistance to sorafenib or TACE. It also provides new insights into the development of more personalized diagnosis and treatment programs for HCC. The substances in exosomes, especially miRNAs, provide a new direction for improving the early diagnosis of HCC. Thus, liquid biopsies can provide researchers with more detailed and personalized information from cancer diagnosis to tumor monitoring by collecting samples continuously. However, there still stands challenges in the way that translates liquid biopsy from bench to bedside. The clinical application of liquid biopsy requires accurate biomarkers and standardized detection methods. In summary, liquid biopsy seems a convenient, noninvasive, and potential method for HCC.

## Author Contributions

J-CY wrote the draft and prepared the tables and figures. J-JH collected the literature. WL designed the tables and figures. Y-XL and J-ZL revised it critically. D-WY designed this review and revised the manuscript. All authors contribute to the article and approved the submitted version.

## Funding

This research was supported by National Natural Science Foundation of China (No. 81873732). This study was funded by Shanxi Province136 Revitalization Medical Project Construction Fund.

## Conflict of Interest

The authors declare that the research was conducted in the absence of any commercial or financial relationships that could be construed as a potential conflict of interest.

## Publisher’s Note

All claims expressed in this article are solely those of the authors and do not necessarily represent those of their affiliated organizations, or those of the publisher, the editors and the reviewers. Any product that may be evaluated in this article, or claim that may be made by its manufacturer, is not guaranteed or endorsed by the publisher.
